# CRISPR/dCas9-Dnmt3a-mediated targeted DNA methylation of *APP* rescues brain pathology in a mouse model of Alzheimer’s disease

**DOI:** 10.1186/s40035-022-00314-0

**Published:** 2022-09-15

**Authors:** Hanseul Park, Jaein Shin, Yunkyung Kim, Takashi Saito, Takaomi C. Saido, Jongpil Kim

**Affiliations:** 1 Laboratory of Stem Cells and Gene Editing, Department of Chemistry & Biomedical Engineering, Dongguk University, Seoul, 100‐715 Republic of Korea; 2 Laboratory for Proteolytic Neuroscience, RIKEN Center for Brain Science, Wako-shi, Saitama, Japan; 3 Department of Neurocognitive Science, Institute of Brain Science, Nagoya City University Graduate School of Medical Sciences, Nagoya, Aichi Japan; 4 Institute for Cellular Rebooting, Dongguk University, Seoul, Korea

**Keywords:** Alzheimer’s disease, *APP*, dCas9, Dnmt3a, Methylation editing

## Abstract

**Background:**

Aberrant DNA methylation patterns have been observed in neurodegenerative diseases, including Alzheimer's disease (AD), and dynamic changes in DNA methylation are closely associated with the onset and progression of these diseases. Particularly, hypomethylation of the amyloid precursor protein gene (*APP*) has been reported in patients with AD.

**Methods:**

In this study, we used catalytically inactivated Cas9 (dCas9) fused with Dnmt3a for targeted DNA methylation of *APP*, and showed that the CRISPR/dCas9-Dnmt3a-mediated DNA methylation system could efficiently induce targeted DNA methylation of *APP* both in vivo and in vitro.

**Results:**

We hypothesized that the targeted methylation of the *APP* promoter might rescue AD-related neuronal cell death by reducing *APP* mRNA expression. The cultured *APP*-KI mouse primary neurons exhibited an altered DNA-methylation pattern on the *APP* promoter after dCas9-Dnmt3a treatment. Likewise, the *APP* mRNA level was significantly reduced in the dCas9-Dnmt3a-treated wild-type and *APP-KI* mouse primary neurons. We also observed decreased amyloid-beta (Aβ) peptide level and Aβ42/40 ratio in the dCas9-Dnmt3a-treated *APP*-KI mouse neurons compared to the control *APP*-KI mouse neurons. In addition, neuronal cell death was significantly decreased in the dCas9-Dnmt3a-treated *APP*-KI mouse neurons. Furthermore, the in vivo methylation of *APP* in the brain via dCas9-Dnmt3a treatment altered Aβ plaques and attenuated cognitive and behavioral impairments in the *APP*-KI mouse model.

**Conclusions:**

These results suggest that the targeted methylation of *APP *via dCas9-Dnmt3a treatment can be a potential therapeutic strategy for AD.

**Supplementary Information:**

The online version contains supplementary material available at 10.1186/s40035-022-00314-0.

## Background

Alzheimer’s disease (AD) is a progressive neurological disease and the most common form of neurodegenerative dementia [1]. At the cellular level, AD is characterized by Aβ accumulation, tau protein hyperphosphorylation, and neuroinflammation [2]. Physiological production of Aβ peptides through amyloid precursor protein (APP) proteolysis by β- and γ-secretases is an essential step in AD pathogenesis [1, 2]. AD is a complex disease that involves many key risk factors [3]. Several studies have suggested the potential role of epigenetic mechanisms in neurodegenerative processes leading to AD [4]. Particularly, changes in epigenetic modification in neuronal cells can trigger variations in gene transcription levels, leading to AD pathogenesis [5]; this highlights the important role of epigenetics in the onset and progression of AD.

Aberrant DNA methylation is associated with neurodegenerative diseases including AD [6, 7]. As an epigenetic mechanism, DNA methylation involves the covalent transfer of a methyl group from S-adenosyl-l-methionine (SAM) to the C-5 position of cytosine (5c) to form 5-methylcytosine (5mC) [8]. This process is mediated by DNA methyltransferase (DNMT) family members, namely, DNMT1, DNMT2, DNMT3a, DNMT3b, and DNMT3l [8, 9]. The catalytic domain of Dnmt3a is the functional domain for CpG methylation. Dnmt3a is a major de novo DNA methyltransferase and predominantly methylates CpG dinucleotides [10]. Also, DNA methylation plays a critical role in many biological processes, including transcription regulation [11], chromosome maintenance [12], and genomic imprinting [13]. In addition, *APP* hypomethylation has been reported in patients with AD, suggesting that DNA methylation may control AD pathogenesis.

Catalytically inactivated Cas9 (dCas9) is an endonuclease-inactivated clustered regularly interspaced short palindromic repeats (CRISPR)/Cas9 mutant, which provides an efficient tool for targeted gene modification [14]. When fused with effector domains, it can be used for various genomic engineering processes, such as DNA methylation [15, 16], gene expression modulation [17], and epigenetic regulation [18]. For instance, the fusion of Dnmt3a or Ten-eleven translocation 1 (Tet1) with dCas9 can induce targeted DNA methylation in the mammalian genome in vitro and in vivo [15]. Targeted DNA methylation around promoter regions can be effectively achieved using dCas9-mediated de/methylation [15, 16]. DNA methylation editing has been used as a novel treatment for a broad range of diseases [19, 20]. For example, dCas9-Dnmt3a-based system has been used to increase DNA methylation for repressing the transcription of oncogenes *CDKN2A* (cyclin-dependent kinase inhibitor 2A) and *BACH2* (BTB domain and CNC homolog 2) in cancer development associated with aberrant DNA methylation [21, 22]. DNA methylation editing has also been implemented in the treatment of several neurodegenerative diseases. For instance, the Cas9-mediated DNA methylation editing of *FMR1* efficiently repairs neuronal abnormalities observed in fragile X syndrome (FXS) [20]. Thus, DNA methylation editing has emerged as a novel approach to controlling gene expression efficiently for treating genetic diseases.

In this study, we used a dCas9-Dnmt3a-mediated DNA methylation editing system to induce *APP* hypermethylation for effectively suppressing APP in primary neurons and in the mouse brain in vivo. Initially, we verified the targeted methylation of the *APP* promoter region by dCas9-Dnmt3a and subsequent suppression of *APP* expression in the mouse brain. Then we examined Aβ42 formation and Aβ-associated memory impairment in the *APP* knock-in (*APP*-KI) mouse model, in order to test the potential of targeted methylation editing of *APP* gene as an AD treatment strategy.

## Methods

### Production of guide RNAs

A single guide RNA (sgRNA) was cloned with a pLenti-sgRNA (Addgene, Cambridge, MA, #71,409) vector. The vector was linearized with the BsmbI (NEB) enzyme at 37 °C for 2 h. Next, guide RNA oligonucleotide pairs were annealed using a T4 polynucleotide kinase (NEB), and 30 ng of the digested pLenti-sgRNA vector was ligated with the annealed oligonucleotides at room temperature (RT) for 2 h. The ligated vectors were transformed, and transformation was confirmed through enzyme digestion and sequence analysis (GACTATCATATGCTTACCGT was used as the primer).

### Animal experiments

All animal experiments were approved by the Institutional Animal Care and Use Committee at Dongguk University and performed in accordance with institutional guidelines. The mice used in this study were maintained under controlled conditions (12-h light/dark cycle at 22–23 °C). *APP*-KI (*App*^*NL-G-F/NL-G-F*^; Swedish [NL], Beyreuther/Iberian [F], and Arctic [G] mutation knock-in) mice were purchased from the RIKEN Brain Science Institute [23]. Three-month-old male *APP*-KI mice and B6C57 mice were used for the experiments. For stereotaxic injection, the male mice were anesthetized with 120 mg/kg Avertin (2,2,2-tribromoethanol; Sigma, St. Louis, MO), and 10 µl of dCas9-Dnmt3a and empty vector (control) or APP -189 sgRNA lentivirus were microinjected into the dentate gyrus (DG) region of each hemisphere at the following coordinates: AP − 2 mm, ML ± 1.1 mm, and DV − 2 mm. One month after injection, the mice were subjected to behavioral tests and biochemical analysis. For the behavioral tests, Y-maze, fear conditioning, and water maze tests were conducted, and observations were recorded and analyzed using Noldus Ethovision XT 13 (Noldus, Netherlands). In the Y-maze test, the number of alternations was determined using three open-arm chambers. The mouse behavior was recorded for 10 min and the total number of spontaneous visits to each arm position was calculated. Fear conditioning tests were conducted for 2 consecutive days. On day 1 of training, the mice were placed in a fear conditioning chamber and allowed to explore for 3 min. Then, they were given pairs of regular stimuli and aversive unconditioned stimuli (1 s, 0.7 mA). After exposure to stimuli, the mice stayed for further 1 min for measurement of freezing behavior. On day 2, freezing behavior tests were conducted to evaluate the conditioned fear, and freezing behavior was recorded for 2 min. The water maze test was performed in a circular water tank. In the visible platform trial, the mice were trained for 4 days, with three trials conducted per day. In the invisible platform test on day 4, the time spent in each quadrant was recorded for 1 min. After the behaviorial test, the 4-month-old male mice were sacrificed for biochemical analysis.

### Cell culture

Mouse primary fibroblasts (MEFs) and NIH/3T3 cells were cultured in Dulbecco's Modified Eagle Medium (Gibco, Waltham, MA) supplemented with heat-inactivated fetal bovine serum (Gibco) and 1% penicillin/streptomycin (Gibco). Primary neurons were obtained from *APP*-KI mice on embryonic day 14 (E14) and maintained in a neurobasal medium (Gibco) supplemented with heat-inactivated fetal bovine serum (Gibco), glutamine (Gibco), B-27™ supplement (Gibco), *L*-glutamine (Gibco), P/S (Gibco), and laminin (Corning®, Corning, NY), for 2 weeks followed by biochemical analysis. All cells were incubated at 37 °C in 5% CO_2_. Cell lines were authenticated by STR (Short Tandem Repeat) analysis (Kogene Biotech, Seoul, Korea), and mycoplasma was tested using a MycoSensor PCR assay kit (Agilent, Santa Clara, CA). For lentivirus production, HEK293T cells were passaged and cultured at 80% confluency and transfected with lentiviral vectors (psPAX2, pMD2.G, and pLenti-sgRNA [Addgene, Cambridge, MA, #71409] or Fuw-dCas9-Dnmt3a [Addgene, #84476]) using calcium phosphate (Sigma) and HEPES (Sigma). The medium was replaced 24 h after transfection, and the virus was harvested by centrifugation after 72 h.

### Off-target analysis

Potential off-target sequences were validated using the off-target site prediction software Cas-OFFinder (http://www.rgenome.net/cas-offinder) [24]. For validation of potential off-target sites, the top six predicted off-target sites with two or less mismatches compared to the on-target sequence were analyzed using quantitative real-time PCR analysis.

### Immunocytochemistry

Cells and hippocampal brain tissues were fixed in 4% paraformaldehyde (Sigma) and washed with PBS buffer. The samples were blocked with PBST supplemented with 1% BSA for 20 min and incubated with anti-NeuN (Invitrogen, PA5-78639), anti-Tuj1 (Sigma, T8578), anti-Map2 (Thermo Fisher Scientific, Waltham, MA, 13-1500), or anti-ab42 (Abcam, Cambridge, UK, ab201060) at 4 °C overnight. Then they were washed with PBST, incubated with appropriate secondary antibodies at RT for 2 h, and counterstained with DAPI (Invitrogen). The stained samples were visualized under an LSM 700 confocal microscope (Zeiss, Oberkochen, Germany). The percentage of Aβ42+/Map2+ cells was calculated as follows: number of Aβ42- and Map2-double positive cells/number of Map2-positive cells × 100%.

### RNA isolation and qRT-PCR

RNA was extracted using an eCube tissue RNA mini kit (Philekorea, Seoul, Korea) in accordance with the manufacturer’s instructions. In brief, 1 μg of the prepared RNA was reverse transcribed using AccuPower® CycleScript RT PreMix (Bioneer, Daejeon, Korea). qRT-PCR was conducted using a Rotor-Gene Q real-time PCR cycler (Qiagen, Hilden, Germany) with suitable primer sets and AccuPower® PCR PreMix (Bioneer, Daejeon, Korea).

### Western blot analysis

The samples (cells and brain tissues) were mixed with radioimmunoprecipitation assay (RIPA) buffer (Sigma), 1 × proteinase inhibitor cocktail (Sigma), and 5 × loading buffer, incubated at 100 °C for 10 min, and centrifuged at 14,000×*g* for 10 min to remove the debris. The prepared samples were separated with SDS-PAGE and blotted onto membranes. The blotted membranes were incubated with anti-APP C-terminal (Sigma, A8717) or anti-β-actin (AbFrontier, Seoul, Korea, LF-PA0207) at 4 °C overnight. They were then incubated with a conjugating secondary antibody. Protein bands were visualized using an ECL kit (Dogen, Seoul, Korea).

### Bisulfite sequencing

Total DNA was extracted using an eCube tissue DNA mini kit (Philekorea, Seoul, Korea), in accordance with the manufacturer’s instructions. In brief, 2 μg of DNA was modified with an EpiTect bisulfite kit (Qiagen, Hilden, Germany) in accordance with the manufacturer’s instructions. The bisulfite-converted DNA was then amplified with PCR primer sets designed via the PrimerSuite website (www.primer-suite.com). The amplified products were extracted using NucleoSpin® gel and a PCR clean-up kit (Macherey–Nagel, Düren, Germany) and cloned using a TA Cloning™ kit (Thermo Fisher Scientific) for sequencing.

### Aβ42 and Aβ40 quantification

Samples (cells and brain tissues) were added with RIPA (Sigma) and  1× proteinase inhibitor cocktail (Sigma), and levels of Aβ40 and Aβ42 were determined using Aβ (1–40) and Aβ (1–42) (FL) kits (IBL International, Hamburg, Germany), respectively. Absorbance at 450 nm was measured using a VERSAmax tunable microplate reader (Molecular Devices, San Jose, CA).

### Statistical analysis

Statistical analyses were conducted using IBM SPSS Statistics (IBM Corp, Armonk, NY). Differences between groups were evaluated using one-way ANOVA and Student’s *t*-test. *P* < 0.05 was considered as statistically significant.

## Results

### Targeted DNA methylation of the *APP* promoter locus using dCas9-Dnmt3a

**Fig. 1:**
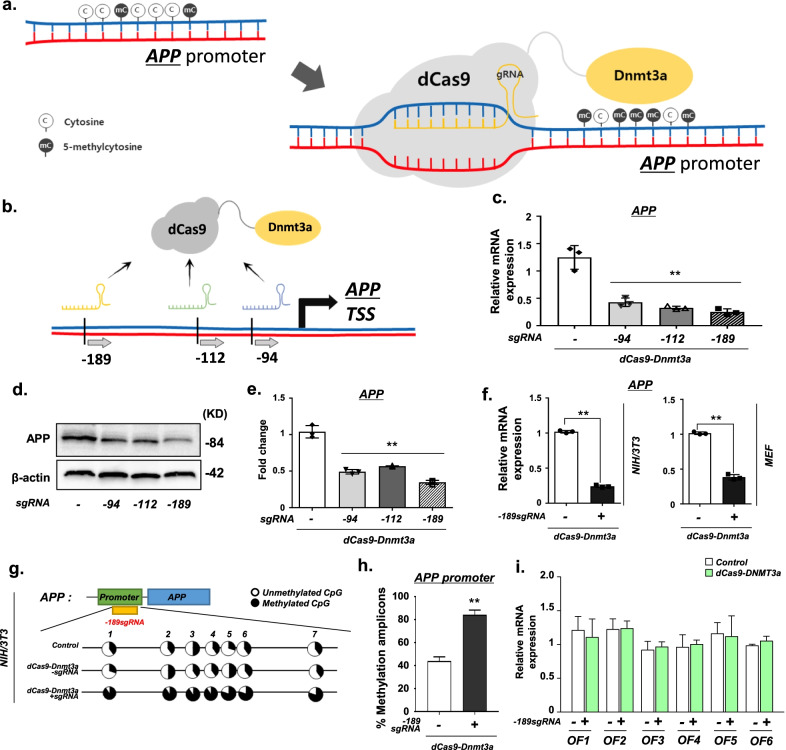
dCas9-Dnmt3a mediated *APP* methylation in vitro*.* (**a**) Schematic of the dCas9-Dnmt3a-mediated *APP* methylation system. **b** Schematic of the location of the *APP* promotor-targeting single guide RNAs (sgRNAs) for *APP* allele methylation. **c** Quantitative real-time PCR analysis of APP mRNA expression with sgRNAs targeting different locations, − 94, − 112, and − 189 bp from the *APP* start codon. Data are expressed as mean ± SEM (*n* = 3). **P < 0.01, one-way ANOVA with Tukey’s *post-hoc* test. **d, e** Western blot analysis of APP N-terminal in NIH/3T3 cells treated with different sgRNAs (− 94, − 112, or − 189 sgRNA). Data are expressed as mean ± SEM (*n* = 3). **P < 0.01, one-way ANOVA with Tukey’s *post-hoc* test. **f** Quantitative real-time PCR analysis of *APP* expression in NIH/3T3 cells and MEF cells treated with − 189 sgRNA. Data are expressed as mean ± SEM (*n* = 3). **P < 0.01, two-sided Student’s *t*-test. **g** Bisulfite sequencing analysis of the promoter region of *APP* in NIH/3T3 cells treated with − 189 sgRNA and dCas9-Dnmt3a. **h** Quantification of methylated amplicons. In 3 independent experiments, 10 to 30 sequencing data were measured in total. Data are expressed as mean ± SEM. **P < 0.01, two-sided Student’s *t*-test. **i** Quantitative real-time PCR analysis of the predicted off-target genes in NIH/3T3 cells treated with − 189 sgRNA and dCas9-Dnmt3a. Data are expressed as mean ± SEM (*n* = 3). *P < 0.05, two-sided Student’s *t*-test. The image in **d** is representative of three or more similar experiments

APP, the precursor of Aβ, plays a pivotal role in the development of AD [2]. Aβ is generated from APP through sequential cleavage by β-secretase, followed by the γ-secretase complex [1, 2]. In the brains of AD patients, aberrant CpG methylation of *APP* occurs, possibly contributing to the aberrant gene expression of *APP*. Targeted DNA methylation editing can be performed by fusing Tet1 or Dnmt3a with dCas9 [15]. Therefore, we evaluated whether the dCas9-Dnmt3a system could be used to alter the methylation level of the *APP* promoter locus (Fig. 1a). Initially, we designed three different sgRNAs targeting the *APP* promoter region at the most efficient binding sites for *APP* methylation (Fig. 1b and Additional file 1: Table S1), and tested the targeting efficacy of these sgRNAs with dCas9-Dnmt3a in NIH/3T3 cells. We found that the *APP* expression in NIH/3T3 cells treated with *APP* sgRNAs and dCas9-Dnmt3a was significantly decreased (Fig. 1c). Particularly, the transduction of the − 189 sgRNA targeting − 189 bp from the start codon of *APP*, with dCas9-Dnmt3a, led to the most effective reduction in *APP* expression (Fig. 1c–e). Thus, dCas9-Dnmt3a coupled with the − 189 sgRNA was used to edit *APP* DNA methylation in the following experiments.

We further confirmed the decrease in *APP* expression in the MEF cell line. The dCas9-Dnmt3a combined with the − 189 sgRNA efficiently decreased the mRNA expression of *APP* in MEF and NIH/3T3 cells (Fig. 1f). Moreover, we validated CpG methylation in the *APP* promoter region by bisulfite sequencing analysis. We observed a significant increase in CpG methylation of the *APP* promoter region in NIH/3T3 cells treated with − 189 sgRNA and dCas9-Dnmt3a (Fig. 1g, h). Additionally, we examined the off-target effects of dCas9-DNMT in treated cells. We analyzed six predicted off-target sites in the NIH/3T3 cells, but none of them were affected by sgRNA and dCas9-Dnmt3a (Fig. 1i and Additional file 1: Table S2). These results indicate that dCas9-Dnmt3a can efficiently edit the methylation of the *APP* promoter and thereby decrease *APP* expression in cultured mouse cells, without any obvious impact on other genes.

### Reduction of Aβ generation via* APP *methylation by dCas9-Dnmt3a in *APP*-KI mouse primary neurons

**Fig. 2:**
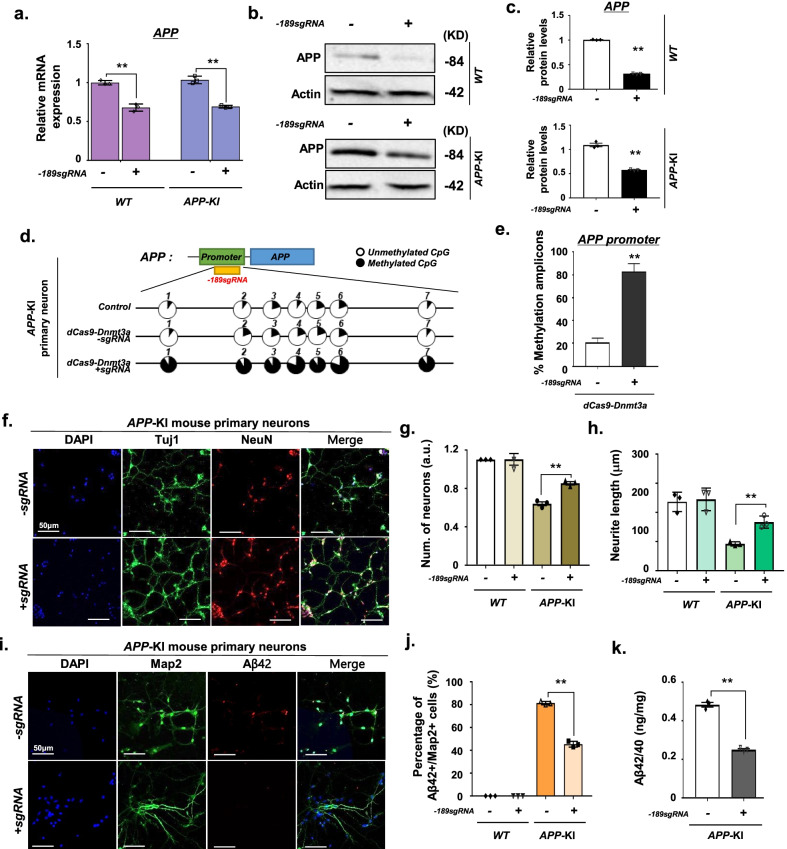
dCas9-Dnmt3a-mediated *APP* methylation enhanced neuroprotection in *APP*-KI mouse primary neurons. **a** Quantitative real-time PCR analysis of *APP* expression in WT and *APP*-KI mouse primary neurons treated with − 189 sgRNA and dCas9-Dnmt3a. Data are expressed as mean ± SEM (*n* = 3). **P < 0.01, two-sided Student’s *t*-test. **b**, **c** Western blot analysis of APP in WT and *APP*-KI mouse primary neurons treated with − 189 sgRNA and dCas9-Dnmt3a. Data are expressed as mean ± SEM (*n* = 3). **P < 0.01, two-sided Student’s *t*-test. **d** Bisulfite sequencing analysis of the promoter region of *APP* in *APP*-KI mouse primary neurons treated with − 189 sgRNA and dCas9-Dnmt3a. **e** Quantification of methylated amplicons. In 3 independent experiments, 10 to 30 sequencing data were measured in total. Data are expressed as mean ± SEM. **P < 0.01, two-sided Student’s *t*-test. **f** Immunostaining of Tuj1 (green), NeuN (red), and DAPI (blue) in *APP*-KI mouse primary neurons treated with − 189 sgRNA and dCas9-Dnmt3a. **g** The number of Tuj1-positive neurons in **f**. Data are expressed as mean ± SEM (*n* = 3). **P < 0.01, two-sided Student’s *t*-test. **h** Quantification of neurite length. In 3 independent experiments, over 100 neurites were measured. Data are expressed as mean ± SEM (*n* = 3). **P < 0.01, two-sided Student’s *t*-test. **i** Map2 (green), Aβ42 (red), and DAPI (blue) immunostaining in *APP*-KI mouse primary neurons treated with − 189 sgRNA and dCas9-Dnmt3a. **j** Percentage of  Aβ42+/Map2+ cells from **i**. In 3 independent experiments, over 100 neurites were measured. Data are expressed as mean ± SEM (*n* = 3). **P < 0.01, two-sided Student’s *t*-test. **k** ELISA of the Aβ42/40 protein ratio in *APP*-KI mouse primary neurons transduced with − 189 sgRNA and dCas9-Dnmt3a. Data are expressed as mean ± SEM (*n* = 3). **P < 0.01, two-sided Student’s *t*-test. The images in **b**, **f**, **i** are representative of three or more similar experiments

We examined whether the dCas9-Dnmt3a-mediated CpG methylation of *APP* could reduce Aβ generation and exert neuroprotection on *APP*-KI mouse primary neurons in vitro. Seven days after transduction of − 189 *APP* sgRNA and dCas9-Dnmt3a into the *APP*-KI mouse primary neurons, we observed a significant decrease in *APP* mRNA expression in the wild-type (WT) and *APP*-KI mouse primary neurons (Fig. 2a). We further verified that transduction of dCas9-Dnmt3a resulted in a significant reduction of APP protein level in the *APP*-KI mouse primary neurons (Fig. 2b, c). In addition, bisulfite sequencing of the promoter region of *APP* revealed that the cytosines of the CpG site at − 189 bp were highly methylated in the dCas9-Dnmt3a-treated primary neurons in which APP expression was suppressed (Fig. 2d, e).

Given that AD is characterized by deterioration of neuronal connectivity or the loss of dendritic spines of pyramidal cells [25, 26], we investigated the effects of dCas9-Dnmt3a-mediated *APP* methylation on neuronal connectivity and dendritic spines. The number of neurons and neurite length decreased in *APP*-KI mouse primary neurons, but these decreases were reversed by dCas9-Dnmt3a transduction (Fig. 2f–h). These results demonstrated that *APP* methylation by dCas9-Dnmt3a and *APP* sgRNA reduced the apoptosis and neurite dystrophy of *APP*-KI mouse primary neurons. Aβ peptide is a marker of AD, promoting the formation of amyloid fibrils that accumulate in senile plaques [1, 27]. Therefore, we determined the Aβ42 levels in primary neurons after transduction with dCas9-Dnmt3a. Results showed that the number of Aβ42+/Map2+ double-positive neurons significantly decreased in *APP-*KI mouse primary neurons transduced with dCas9-Dnmt3a (Fig. 2i, j). We also validated the decrease in the Aβ42/Aβ40 ratio in the dCas9-Dnmt3a-infected *APP-*KI mouse primary neurons (Fig. 2k). Our results suggest that *APP* methylation via dCas9-Dnmt3a can alleviate neuronal deficits in *APP-*KI mouse primary neurons.

### In vivo dCas9-Dnmt3a-mediated *APP* methylation in the brain

**Fig. 3:**
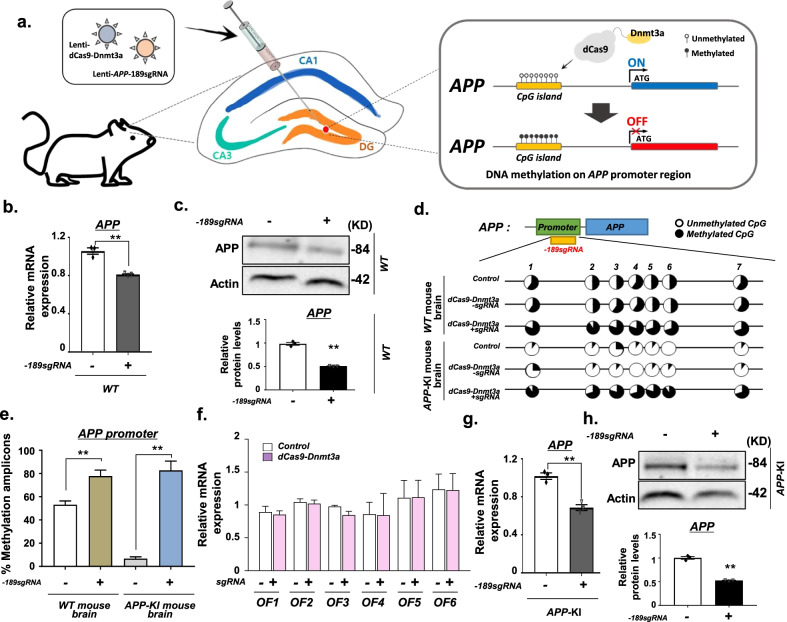
dCas9-Dnmt3a-mediated DNA methylation diminished *APP* expression in mouse brain. **a** Schematic of DNA methylation editing in the *APP* target region in the mouse brain in vivo. **b** Quantitative real-time PCR analysis of *APP* expression in the wild-type (WT) mouse brain infected with − 189 sgRNA and dCas9-Dnmt3a. Data are expressed as mean ± SEM (*n* = 3). **P < 0.01, two-sided Student’s *t*-test. **c** Western blot analysis of APP expression in the WT mouse brain transduced with − 189 sgRNA and dCas9-Dnmt3a. Data are expressed as mean ± SEM (*n* = 3). **P < 0.01, two-sided Student’s *t*-test. **d** Bisulfite sequencing analysis of the *APP* promoter region from the WT mouse brain and *APP*-KI mouse brain infected with − 189 sgRNA and dCas9-Dnmt3a. **e** Quantification of methylated amplicons. In 3 independent experiments, 10 to 30 sequencing data were measured in total. Data are expressed as mean ± SEM. **P < 0.01, two-sided Student’s *t*-test. **f** Quantitative real-time PCR analysis of predicted off-target genes in the WT mouse hippocampus transduced with − 189 sgRNA and dCas9-Dnmt3a. Data are expressed as mean ± SEM (*n* = 3). *P < 0.05, two-sided Student’s *t*-test. **g** Quantitative real-time PCR analysis of *APP* expression in the *APP*-KI mouse hippocampus transduced with − 189 sgRNA and dCas9-Dnmt3a. Data are expressed as mean ± SEM (*n* = 3). **P < 0.01, two-sided Student’s *t*-test. **h** Western blot analysis of APP in the *APP-*KI mouse hippocampus transduced with − 189 sgRNA and dCas9-Dnmt3a. Data are expressed as mean ± SEM (*n* = 3). **P < 0.01, two-sided Student’s *t*-test. The images in **c**, **h** are representative of three or more similar experiments

To evaluate whether dCas9-Dnmt3a-mediated methylation editing could be used for in vivo *APP* methylation, we injected the lentivirus containing dCas9-Dnmt3a with *APP* sgRNA directly into the DG of mouse brain (Fig. 3a). The transduction of dCas9-Dnmt3a significantly decreased the mRNA expression and protein level of APP in the WT mouse brain (Fig. 3b, c).

We also tested whether dCas9-Dnmt3a could increase the level of *APP* methylation in the brain. Bisulfite sequencing showed that the demethylated CpG sites in the *APP* promoter were extensively methylated by dCas9-Dnmt3a (Fig. 3d, e). To further investigate the potential in vivo off-target effects of dCas9-Dnmt3a in the brain, predicted off-target sites were tested (Fig. 3f). The results indicated that the − 189 *APP* sgRNA had no off-target effects (Fig. 3f). Additionally, we verified the effect on *APP* expression levels in *APP*-KI mice. After injection of dCas9-Dnmt3a with *APP* sgRNA into the *APP*-KI mouse brain, *APP* expression diminished to a similar level as that in the WT mouse brain (Fig. 3g, h). These findings indicate that dCas9-Dnmt3a induces efficient DNA methylation editing in the brain.

### Improvement of cognitive deficits in the *APP*-KI mouse model through *APP* methylation editing by dCas9-Dnmt3a

**Fig. 4:**
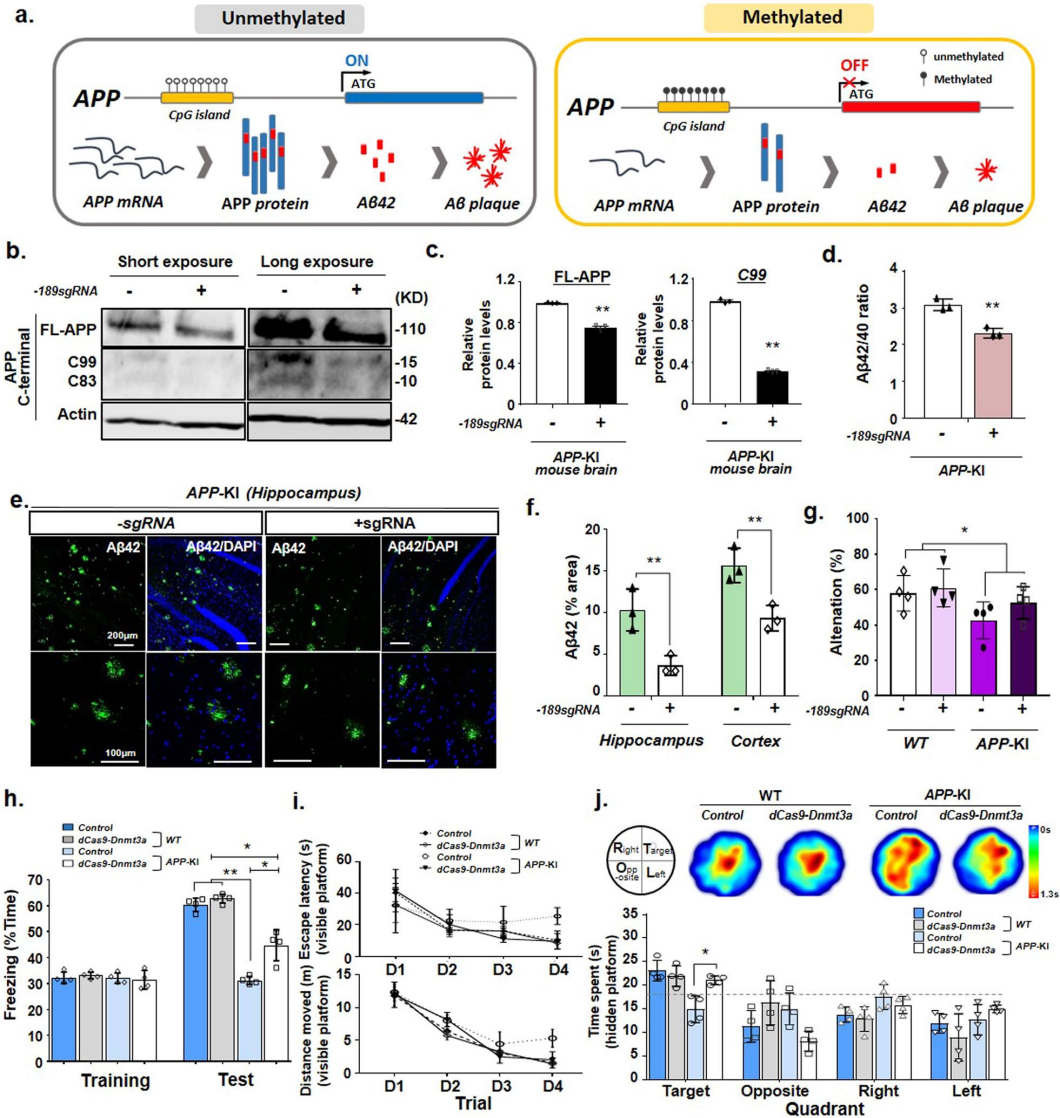
dCas9-Dnmt3a-mediated DNA methylation elicited therapeutic effects on the *APP-*KI mouse model. **a** Schematic of a DNA methylation editing-based strategy for the *APP-*KI mouse model. **b**, **c** Western blot analysis and quantification of full-length APP (FL-APP), β-secretase-derived C-terminal fragment (CTF) (C99), γ-secretase-derived CTF (C93), and beta-actin (Actin) in the *APP-*KI mouse hippocampus infected with − 189 sgRNA and dCas9-Dnmt3a. Data are expressed as mean ± SEM (*n* = 3). **P < 0.01, two-sided Student’s *t*-test. **d** ELISA analysis of the Aβ42/40 ratio in the *APP-*KI mouse hippocampus transduced with − 189 sgRNA and dCas9-Dnmt3a. Data are expressed as mean ± SEM (*n* = 3). **P < 0.01, two-sided Student’s *t*-test. **e** Aβ42 (green) and DAPI (blue) immunostaining in the *APP-*KI mouse hippocampus transduced with − 189 sgRNA and dCas9-Dnmt3a. **f** Quantification of the Aβ42 area presented in **e**. Data are expressed as mean ± SEM (*n* = 3). **P < 0.01, two-sided Student’s *t*-test. **g** Y-maze spontaneous alternation of WT and *APP*-KI mice transduced with − 189 sgRNA and dCas9-Dnmt3a. *P < 0.05; *P* > 0.05 *APP*-KI vs *APP*-KI+ −189 dCas9-Dnmt3a group, one-way ANOVA with Tukey’s *post-hoc* test. **h** Percentage of freezing behavior during the contextual fear memory test. Data are expressed as mean ± SEM. *P < 0.05, **P < 0.01, one-way ANOVA with Tukey’s *post-hoc* test. **i**, **j** Morris water maze test for evaluation of long-term spatial memory. **i** Escape latency (upper) and total distance moved (lower) during the visible platform training session. Data are expressed as mean ± SEM. *P < 0.05, one-way ANOVA with Tukey’s *post-hoc* test. **j** Heatmap and quantification represent the quadrant occupancy time during the hidden platform test session. Data are expressed as mean ± SEM. *P < 0.05, one-way ANOVA with Tukey’s *post-hoc* test. The images in **b**, **e** are representative of three or more similar experiments

Aβ accumulation is associated with neurodegeneration and cognitive decline [2]. Thus, we tested whether *APP* methylation via dCas9-Dnmt3a in the mouse brain could decrease Aβ (1–42) and reduce cognitive and memory impairments in the *APP*-KI mouse model. We injected dCas9-Dnmt3a into the DG region of the *APP*-KI mouse brain and conducted biochemical and behavioral analyses 4 weeks after injection (Fig. 4a). Results showed decreased *APP* expression in the DG of dCas9-Dnmt3a-injected *APP*-KI mice compared with that in the DG of age-matched *APP*-KI control mice (Fig. 3g, h). We validated the methylation range at the *APP* promoter locus in the brain through bisulfite sequencing, and found that the demethylated CpG sites in the *APP* promoter were extensively methylated by dCas9-Dnmt3a compared with those in age-matched *APP*-KI control mice (Additional file 1: Fig. S1a, b).

To investigate the effects of *APP* methylation via dCas9-Dnmt3a on AD pathologies, we examined Aβ formation in the *APP*-KI mouse model. Four weeks after injection, dCas9-Dnmt3a significantly reduced the level of full-length APP compared with that in the age-matched *APP*-KI control mice. In addition, the level of β-secretase-derived APP C-terminal fragment, specifically C99, which generates Aβ peptides, was decreased in the dCas9-Dnmt3a-treated *APP*-KI mice (Fig. 4b, c; Additional file 1: Fig. S2). Moreover, dCas9-Dnmt3a significantly reduced the Aβ42/40 ratio in the hippocampus of *APP*-KI mice (Fig. 4d). Consistent with these results, Aβ42 plaque accumulation in the hippocampus and cortex of *APP*-KI mice was significantly decreased by dCas9-Dnmt3a (Fig. 4e, f). These results indicate that the dCas9-Dnmt3a-mediated *APP* methylation attenuated *APP* expression, resulting in reduced Aβ levels in *APP*-KI mice.

Next, behavioral tests were performed to determine whether cognitive behaviors were affected by *APP* methylation via dCas9-Dnmt3a. First, the Y-maze test was performed to evaluate the spatial working memory of *APP*-KI mice. The percentage of alternation decreased in the *APP*-KI mice compared to the WT mice,  but there was no significant difference between the dCas9-Dnmt3a-injected *APP*-KI mice and the age-matched *APP*-KI control mice (Fig. 4g). Next, short-term memory was examined using the contextual fear conditioning test. Interestingly, after 24 h of training, the freezing levels of the dCas9-Dnmt3a-injected *APP*-KI mice were significantly increased (Fig. 4h). To further investigate the spatial reference memory and working memory, the Morris water maze test was performed. The escape latency and distance of movement showed no significant difference between the control and dCas9-Dnmt3a-injected groups in the visible platform trials (Fig. 4i). In the test without the platform, the dCas9-Dnmt3a-injected *APP*-KI mice spent significantly more time in the target quadrant than the age-matched *APP*-KI control mice (Fig. 4j). These findings showed that dCas9-Dnmt3a prevented decline in learning ability and improved memory retention in *APP*-KI mice.

## Discussion

AD is characterized by progressive cognitive decline [1], with pathologic manifestations of neurofibrillary tau tangles and amyloid plaque accumulation, which are responsible for the gradual deterioration of cognitive ability [1, 28]. Current AD medications help temporarily alleviate AD symptoms [29]. Cholinesterase inhibitors are the only drugs that can greatly improve memory functions of AD patients to date [29, 30]. Some medications can improve behavioral symptoms of AD; however, they are associated with an increased fatality rate in older patients with AD experiencing progressive neuronal degeneration [30]. Consequently, alternative treatments for controlling AD are being explored.

Aβ peptide production through APP proteolysis is an essential step in AD pathogenesis [2]. *APP* hypomethylation, which increases Aβ peptide production, has also been reported in patients with AD [5, 31]. However, deletion of *APP*, *APLP1*, and *APLP2* can cause abnormal cortical migration, suggesting that APP also participates in synaptic pruning and neural outgrowth [32]. Therefore, knockout of these genes is not an ideal strategy for AD treatment. Thus, epigenetic alterations of *APP* can be an alternative strategy for AD treatment. In this study, we demonstrated that the CpG methylation of the *APP* promoter by dCas9-Dnmt3a decreased the generation of neurotoxic Aβ peptides and improved AD-associated learning and memory impairment. The dCas9-Dnmt3a treatment also led to the alteration of *APP* methylation in mouse brain with minimal off-target effects; this result suggests the potential of dCas9-Dnmt3a-mediated *APP* hypermethylation for AD treatment. More importantly, our results indicate that aberrant *APP* CpG methylation can be involved in AD pathogenesis. In individuals aged ≥ 70 years, *APP* is demethylated and excessively overexpressed, ultimately producing Aβ peptides [22, 31, 33]. Therefore, regulation of DNA methylation plays an important role in the inhibition of AD pathogenesis.

Therapeutic epigenetic editing approaches have promising applications as innovative treatments for many neurodegenerative diseases [20, 34, 35]. For example, aberrant DNA methylation, including aberrant hypomethylation and hypermethylation, plays key roles in the pathogenesis of FXS or amyotrophic lateral sclerosis (ALS) [20, 34]. DNA methylation in *FMR1* or *C9orf72* promoter region elicits protective effects against pathologies observed in FXS and ALS [20, 34]. Moreover, de novo DNA methylation of the alpha-synuclein gene has potential for the treatment of Parkinson’s disease (PD) [35]. Thus, by combining different epigenetic effector domains, such as DNMT3 and Tet1, with dCas9 [22, 36], therapeutic epigenetic editing acts as a promising therapeutic tool for various neurological diseases, including FXS, ALS, and PD, with minimal side effects. Epigenetic editing induces reversible changes in DNA sequence, whereas gene editing causes irreversible changes; consequently, epigenetic editing has considerable advantages over gene editing for applications in a clinical setting [36]. However, several aspects of epigenetic editing, including off-target effects and long-term safety, need to be addressed before clinical application. Also, the faithful dCas9-mediated transmission for long-lasting and durable therapeutic effects is essential for achieving more effective and safer therapeutic effects.

CRISPR-based gene editing strategies hold promise for the treatment of AD [37–41]. However, numerous issues need to be addressed before clinical application of epigenetic editing-based therapies in AD patients. For example, clinical trials integrating viral vectors with the CRISPR/Cas9 system can lead to random insertional mutagenesis. To address this issue, non-integrating viruses or lipid vesicles need to be developed for efficient delivery of CRISPR/Cas9 components. Also, injection of viruses for delivery of dCas9-Dnmt3a could elicit immune responses. Thus, studies on the safety of this strategy are required prior to application in human patients. Both effectiveness and safety of dCas9-Dnmt3a delivery systems need to be improved, in order to accelerate the wide use of CRISPR/Cas9-based genome editing strategies for gene editing and repair in the future. Moreover, we found that injection of dCas9-Dnmt3a before plaque deposition effectively modulated AD pathogenesis in the AD mouse model (Fig. 4b–f); such epigenetic editing may effectively reduce the risk of AD or delay the onset of more severe symptoms before the formation of plaques and symptoms. However, since *APP* epigenetic editing can reduce the amount of Aβ peptides by preventing *APP* transcription in the brain, *APP* epigenetic editing could slow down AD progression or stop the destruction of nerve cells even after plaque formation. Thus, to achieve the best outcome, it is important to optimize the time point of CRISPR-based epigenetic editing in AD patients. Also, the persistence of epigenetic editing effects needs to be evaluated. Here, we observed that the dCas9-Dnmt3a-mediated CpG methylation at the *APP* promoter was maintained up to 4 weeks after initial injection (Additional file 1: Fig. S1a). Further studies examining longer persistence of epigenetic editing effects, e.g., for more than 6 months or years, are needed.

## Conclusions

In summary, our study established a novel AD treatment strategy through the regulation of *APP* DNA methylation using dCas9-Dnmt3a. Thus, the CRISPR/dCas9-mediated epigenetic editing offers a highly efficacious targeted epigenome engineering approach for future clinical applications in precision medicine.

## Supplementary Information

**Additional file 1: Table S1.** sgRNA sequences targeting *APP*. **Table S2.** Predicted off-target sites for − 189 sgRNA activity. **Fig. S1** Bisulfite sequencing analysis of the methylation status of the *APP* promoter in the hippocampus of *APP*-KI mice. **Fig. S2** Full scans of the western blots shown in Fig. 4b.

## Data Availability

Not applicable.

## Abbreviations

AD: Alzheimer's disease
APP: Amyloid precursor protein
Aβ: Amyloid-beta
dCas9: Catalytically inactivated Cas9
DG: Dentate gyrus
DNMT: DNA methyltransferase
5c: C-5 position of cytosine
5mC: 5-Methylcytosine
