# Supplementary material for: CRISPR/dCas9-Dnmt3a-mediated targeted DNA methylation of APP rescues brain pathology in a mouse model of Alzheimer’s disease
Source: Transl Neurodegener. 2022 Sep 15;11:41. doi: 10.1186/s40035-022-00314-0 (PMC9476342; doi:10.1186/s40035-022-00314-0)
Supplement: Supplementary file 1 — Additional file 1: Table S1. sgRNA sequences targeting APP. Table S2. Predicted off-target sites for − 189 sgRNA activity. Fig. S1 Bisulfite sequencing analysis of the methylation status of the APP promoter in the hippocampus of APP-KI mice. Fig. S2 Full scans of the western blots shown in Fig. 4b. [file 40035_2022_314_MOESM1_ESM.pdf]

Supplementary Table S1. sgRNA sequences targeting *APP*.

Target	Sequence	Location	Strand
APP	ACCGGGAGCAGAGCGAGCGCGGGG	-94	+
APP	GCGGCGGGAGGCGAGAGCACCGGG	-112	+
APP	GGCGGGATCAGCTGACTCTGCCGG	-189	+
APP	AGCTCCCCGAGGCTCCGCTAGGGG	-228	+

Protospacer adjacent motif sequences are underlined in red.

Supplementary Table S2. Predicted off-target sites for −189 sgRNA activity.

No.	Target	Chromosome	Position	Direction
ON	GGCGGGATCAGCTGACTCTGCCGG	chr16	85173738	-
OF1	GGCGGGgTCAGCTGCCACTCTGaGGG	chr19	41105854	-
OF2	G--GaGATCAGgTGACTCTGCTGG	chr9	44249405	-
OF3	GGCGGGATC--CTGcCTCTGtGGG	chr9	58076622	-
OF4	Ga-GGGATCtGCTGACTCTGCAGG	chr5	30423430	+
OF5	GGCGGGgTCAG—GACTCcGCAGG	chr17	63936803	-
OF6	GGCtGGAgCAGCTG--TCTGCAGG	chr13	46525464	+

Off-target sites were predicted as those having no more than two mismatches compared to the on-target sequence. ON, on-target; OF, off-target.

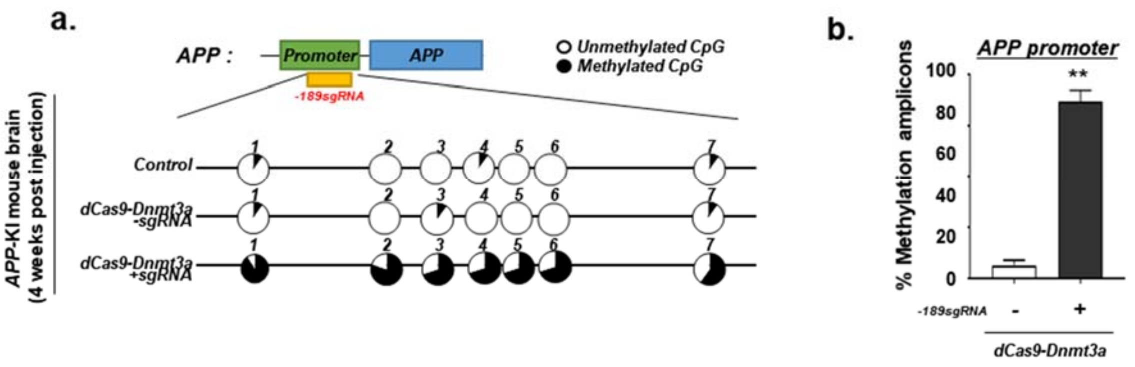

Supplementary Figure S1. Bisulfite sequencing analysis of the methylation status of the *APP* promoter in the hippocampus of *APP*-KI mice. (**a**) Bisulfite sequencing analysis of the promoter region of *APP* in the *APP*-KI mouse hippocampus transduced with −189 sgRNA and dCas9-Dnmt3a 4 weeks after injection. (**b**) Quantification of methylated amplicons. Data are expressed as mean ± SEM (n = 3). **P < 0.01, two-sided Student's t-test.

Figure 4b

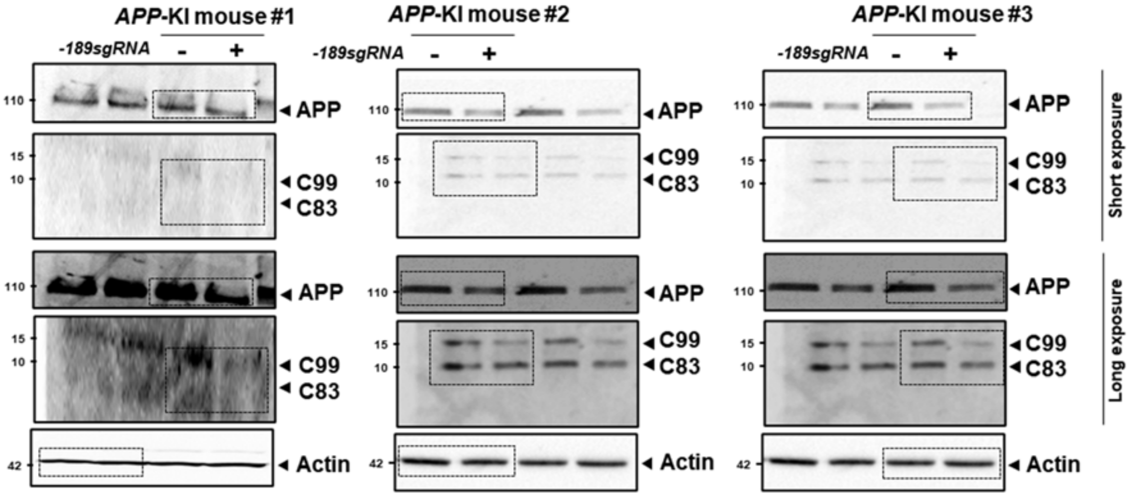

Supplementary Figure S2. Full scans of the western blots shown in Fig. 4b. Blots for all three independent experiments are shown.
